# Effect of acupuncture and its influence on visceral hypersensitivity in IBS-D patients: Study protocol for a randomized controlled trial

**DOI:** 10.1097/MD.0000000000010877

**Published:** 2018-05-25

**Authors:** Lixia Pei, Hao Chen, Jing Guo, Lu Chen, Xiaoliang Wu, Wanli Xu, Shengjie Weng, EunMee Yang, Trine Hammer, Jianhua Sun

**Affiliations:** a Department of acupuncture, Jiangsu Province Hospital of Traditional Chinese Medicine, Affiliated Hospital of Nanjing University of Traditional Chinese Medicine, Hanzhong Road, Qinhuai District; b Nanjing University of Traditional Chinese Medicine, Xianlin Road, Qixia District, Nanjing, China.

**Keywords:** acupuncture, IBS-D, Micro-RNA199, randomized controlled trial, study protocol, TRPV1, visceral hypersensitivity

## Abstract

**Background:**

Irritable bowel syndrome (IBS) is a common functional gastrointestinal disorder associated with visceral hypersensitivity. Increased expression of colonic TRPV1 and decreased expression of microRNA-199 are implicated in the pathogenesis of visceral hypersensitivity in IBS-D patients. Acupuncture is one of the frequently used complementary and alternative therapies for the treatment of IBS. The existing clinical studies mostly use IBS-SSS or other subjective scales, so there is a lack of objective biochemical evidence regarding the effect of acupuncture on IBS. Therefore, we designed this study to investigate whether acupuncture alleviate visceral hypersensitivity by influencing the expression of TRPV1 and microRNA-199.

**Method:**

This study is a randomized, sham-controlled trial involving 40 patients and 10 healthy volunteers. A total of 40 eligible patients with IBS-D will be randomly assigned to a traditional acupuncture group or sham acupuncture group in a 1:1 ratio. Patients will receive 3 acupuncture treatment sessions per week for 12 consecutive weeks, for a total of 36 sessions during the study. The primary outcome measure is the IBS-Symptom Severity Score (IBS-SSS). Secondary outcomes are Visceral Pain Scale and levels of TRPV1 and microRNA-199 in colonic tissues. Healthy volunteers will not receive any clinical intervention. The safety of interventions will be assessed at every visit.

**Discussion:**

The purpose of this trial is to evaluate the efficacy of acupuncture for IBS-D through IBS-SSS and Visceral Pain Scale. Furthermore, we want to explore the intervention mechanism of acupuncture in improving visceral hypersensitivity by analyzing the colonic TRPV1 and microRNA-199.

**Trial registration:**

This trial is registered with Chinese Clinical Trials Register, ChiCTR-IOR- 17010860(http://www.chictr.org.cn/showproj.aspx?proj=18445).

## Abstract 2

Supplemental Digital Content is available in the text

## 1 Introduction

Irritable bowel syndrome (IBS) is a functional gastrointestinal disorder characterized by abdominal pain associated with stool abnormalities and changes in stool consistency. IBS adversely impacts the patients’ quality of life, mental health, and social interaction, imposing significant burden not only on the patients, but also on their families and the public healthcare system. The population prevalence of IBS is high, with a pooled global prevalence of 11.2% (95%CI:9.8–12.8).^[1]^ As one subtype of IBS, IBS-D (diarrhea predominant IBS) accounts for about one-third of the affected population.^[2]^ The treatment strategy of IBS-D includes antispasmodics for abdominal pain, antidiarrhoeals, nutritional interventions and psychotherapy.^[3]^ At present, pharmacological therapies are mainly used for symptomatic relief and do not effectively address the underlying cause of IBS. Moreover, short-term application is often ineffective, while long-term use has side effects. So there is a certain degree of difficulty in treating IBS-D due to a lack of effective therapy.

IBS is a multifactorial disease, with a complex underlying pathogenesis. Several functional alterations have been described, such as enhanced visceral sensitivity, functional brain alterations, bowel motility and secretory dysfunctions, and somatic and psychiatric comorbidities.^[4]^ In recent research, there has been a growing scientific interest in studying the role of visceral hypersensitivity in the pathogenesis of IBS-D. Visceral hypersensitivity refers to an increased intestinal perception, whereby an otherwise physiologic stimuli are perceived as discomfort and pain.^[5]^ It is associated with up-regulation and sensitization of transient receptor potential cation channels (TRPV) in the peripheral sensory neurons.^[6]^ The sensory neurons expressing TRPV are found throughout the digestive tract in the myenteric ganglia and produce pain and/or burning sensation when activated by capsaicin, heat, acid, and inflammatory mediators.^[7]^ Several researches have demonstrated that increased TRPV1 nerve fibers are observed in IBS and may contribute toward the development of visceral hypersensitivity and pain in IBS.^[8,9]^ MicroRNA-199 (miR-199) is a key factor in the regulation of TRPV1. Colonic miR-199 is decreased in patients with IBS-D with visceral pain and directly correlates with increased colonic TRPV1 expression and visceral pain scores.^[10]^ Electroacupuncture (EA) has been shown to alleviate colorectal hypersensitivity and may correlate with the regulatory mechanism of TRPV1.^[11]^

As one of the main components of traditional Chinese medicine (TCM), acupuncture has been frequently used in the treatment of IBS.^[12]^ According to a recent meta-analysis, the effect of acupuncture in controlling IBS symptoms was both clinically and statistically significant.^[13]^ Acupuncture has been shown to lower the severity and frequency of abdominal pain, diarrhea, abdominal distension, and increase the quality of life (QOL) in patients with IBS.^[14]^ However, while research shows that acupuncture can improve IBS symptoms, its effect on objective outcomes is not yet clear. In most previous clinical trials, the main outcome measures were subjective evaluation scales, such as IBS-Severity Scoring System (IBS-SS), IBS-Quality of Life (IBS-QOL), and Hamilton Rating Scale for Anxiety (HAMA). While these are valuable tools for assessing IBS-related symptoms, they are subjective and do not provide objective evidence to support the effect of a given treatment.

Given the current lack or shortage of objective data regarding the efficacy of acupuncture in treating IBS-D, we have chosen to include miR-199 and TRPV1 in the outcome measures of this study. Hence, we hypothesize that acupuncture alleviates visceral hypersensitivity in IBS-D patients by increasing the expression of miR-199 in the colon and reducing the activation of TRPV1. This study is intended to further verify the effect of miR-199 and TRPV1 on visceral hypersensitivity and their roles in the pathogenesis of IBS. Altogether, we will compare the colon miR-199 and TRPV1 expression before and after the treatment, in order to verify the hypothesis and ultimately provide new scientific basis for revealing the mechanism of acupuncture in the treatment of IBS.

## 2 Methods

### 2.1 Trial design

**Figure 1:**
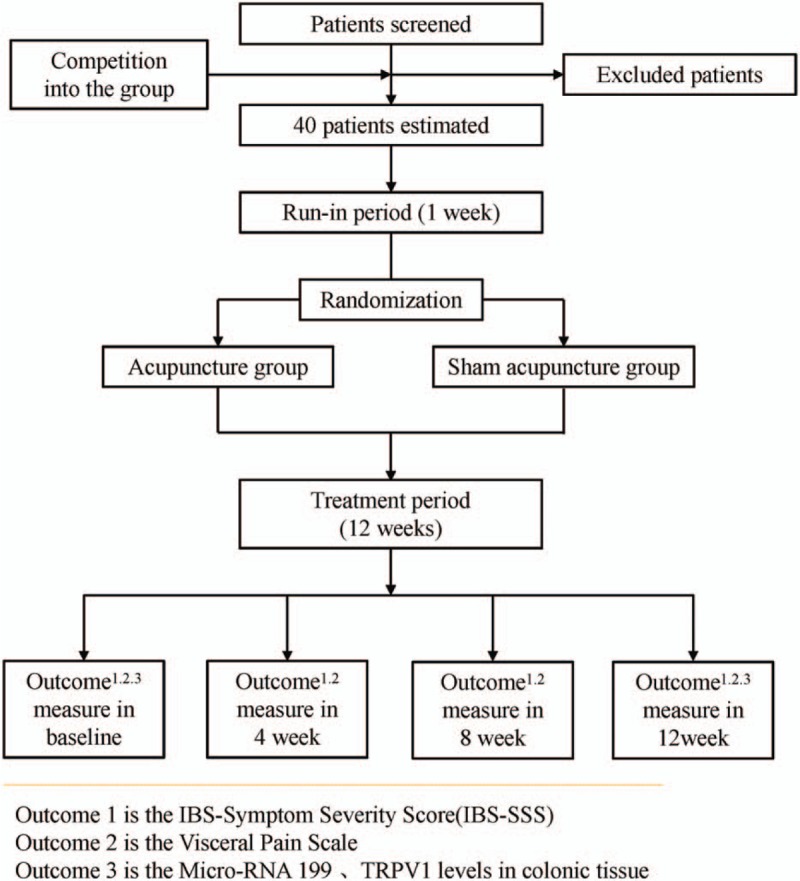
Participant flow diagram.

A single-center randomized controlled trial (RCT) has been designed to compare the effect of traditional acupuncture and sham acupuncture in the treatment of IBS-D patients and regulation of colon miR-199 and TRPV1. The clinical trial conforms to the Consolidated Standards of Reporting Trials (CONSORT 2010) guidelines^[15]^ as well as to the Standards for Reporting Interventions in Controlled Trials of Acupuncture (STRICTA).^[16]^ In total, 40 patients with IBS-D and 10 healthy volunteers will be enrolled. According to the study plan, patients will receive 10 treatments over 12 weeks. This trial will be conducted in Jiangsu Province Hospital of TCM from January 2017 to December 2018. Figure 1 shows the trial procedure and Table 1 details the trial schedule.

**Table 1:**
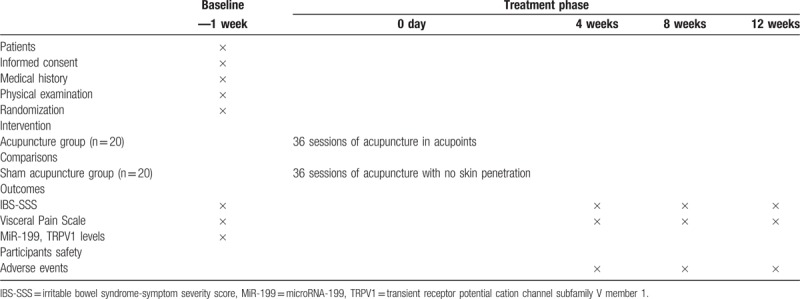
Schedule of enrolment, interventions, and assessments.

### 2.2 Setting

A total of 40 patients diagnosed with IBS-D will be divided randomly into 2 groups (acupuncture group and sham acupuncture group) through “Sample” program based on “R” statistical analysis software in a ratio of 1:1. Ten healthy volunteers will also be enrolled to form a healthy control group. This RCT will be conducted in a single center with the assessor and statistician blinded to treatment allocation. The study will be carried out in Jiangsu Province Hospital of TCM, Nanjing, Jiangsu, China.

### 2.3 Ethics and registration

Our protocol complies with the principles of the Declaration of Helsinki and has been approved by the central Independent Ethics Committee (IEC) of Jiangsu Province Hospital of Traditional Chinese Medicine (TCM) for the various centers (reference number: 2016NL-078-03) (Ethical approval is shown in supplementary materials as *Supplementary 1*). We have also registered at Chinese Clinical Trials Register(ChiCTR-IPR-15007127, URL: http://www.chictr.org.cn/showprojen.aspx?proj=11989). The overall supervision of our trial will be under the charge of the central IEC of the Jiangsu Province Hospital of TCM; any change in the protocol will be submitted to and decided by the Ethics Committee.

## 3 Participants

### 3.1 Diagnostic criteria

*IBS*: IBS will be diagnosed as recurrent abdominal pain or discomfort at least 1 day per week in the last 3 months associated with 2 or more of the following: improvement with defecation; onset associated with a change in stool frequency; and onset associated with a change in stool form (appearance).^[17]^

*IBS-D*: IBS-D subtype will be diagnosed according to the Bristol Stool Chart and Bristol Stool Scale, which classifies IBS-D with the following stool form and consistency: >25% of the defecation are loose and watery (Bristol types 6 and 7) and < 25% of the defecation are hard and lumpy (Bristol types 1 and 2).^[17]^

### 3.2 Inclusion criteria

Patients meeting the following criteria will be selected as study volunteers. The inclusion criteria for IBS-D patients will include: patients meeting the Rome IV diagnostic criteria for IBS-D, men and women aged between 18 and 70 years, the absence of morphological changes and biochemical abnormalities, baseline IBS-SSS score ≥ 75, no medicine therapy for IBS-D (except for emergency) within at least 2 weeks and no acupuncture therapy within 3 months prior to study recruitment, willingness to participate in the study and be randomly allocated into study groups.

### 3.3 Exclusion criteria

Patients meeting any of the following criteria will be excluded from the study: intestinal organic diseases or systemic diseases affecting gastrointestinal motility (such as gallbladder pancreatitis, hyperthyroidism, diabetes, chronic renal insufficiency, nervous system diseases), other chronic diseases including cardiac disease, liver, or kidney disease, thrombocytopenia with bleeding tendency, a severe psychiatric or psychological disorder, history of abdominal or rectal anus surgery, pregnancy or breast feeding, and post-partum ≤12 months, installation of cardiac pacemaker, metal allergy, and fear of the needle, any other condition that the investigators judge as likely to make the patient incapable to complete, comply, or unsuitable for the clinical trial.

### 3.4 Recruitment

There will be 3 primary strategies to recruit participants with IBS-D. The first strategy is to recruit participants who are patients in the Department of Acupuncture, Department of Gastroenterology, and Department of Digestive Endoscopy in Jiangsu Province Hospital of TCM. Second, printed recruitment posters will be distributed in public clinics to enroll potential eligible study subjects. Third, we will post on-line advertisements to briefly introduce our study and recruit patients who are willing to participate.

### 3.5 Randomization/allocation

All eligible participants will be randomized into the acupuncture group or sham acupuncture group, in a 1:1 ratio, using “Sample” program based on “R” statistical analysis software. The randomization grouping code will be placed inside sealed and opaque envelopes and offered after the recruitment of the eligible patients, so that allocation concealment is ensured.

### 3.6 Blinding

The participants as well as research assistants and statisticians assessing the outcome measures will be blinded to treatment allocation. The location of the acupuncture points and manipulation of needles are similar in the 2 groups, thereby optimizing the blinding of subjects. All participants will be asked to guess whether they have received traditional acupuncture or sham acupuncture within 5 minutes after one of the treatment sessions in the week 12 to assess the blinding. However, the acupuncturists providing the interventions cannot be blinded as they will perform either traditional or sham acupuncture.

### 3.7 Informed consent

The details of our study, including trial objectives, characteristics, probable benefits and risks, other available treatment alternatives, and the subjects’ rights as well as obligations as stated in the Declaration of Helsinki, will be made clear to the patients. After obtaining their written informed consent, the subjects will be enrolled in the study. During the process of the trial, if new points regarding the study ethics emerge, the informed consent will be revised and re-submitted to the Ethics Committee, and after approval, the participants’ informed consent will be requested again. In case of the patients’ withdrawal, their available data will be kept for the final analyses.

### 3.8 Safety monitoring

After recruitment and before randomization, all participants will receive routine blood and stool tests as well as examinations for liver and kidney functions in order to identify and exclude those who have severe heart, liver, and/or kidney diseases. The participants will receive these examinations again at the end of study to evaluate any possible side effects of the interventions. Possible adverse events due to acupuncture, such as fainting, needle sticking, local infections, and subcutaneous hematoma, will be properly addressed, analyzed, and documented by the research staff at every session. Any serious adverse event associated with the trial will be reported to the principal investigator immediately. All other unexpected and unintended responses will also be documented as adverse events by the researchers, even if they are not necessarily related to the acupuncture intervention.

### 3.9 Quality control

All practitioners, including acupuncturists, research assistants, and statisticians, will be required to attend training to ensure the quality of this trial. All acupuncturists in this trial have completed their professional training in acupuncture in universities of Chinese medicine and have more than 2 years of clinical experience. The interventions will be performed based on rigorous adherence to the standardized operating procedure.

In order to standardize clinical operations and deliver clinical quality assurance, we developed a series of documents and standardized clinical management operating specifications. Developing appropriate standard operating norms for various stages of clinical research is a way to ensure homogeneity between various researchers. It is helpful to use file management and develop a standard operating procedure (SOP) to ensure the feasibility, safety, and scientific integrity of clinical research.

## 4 Interventions

### 4.1 Acupuncture group

The acupuncture points are identified in accordance with the method of point location issued by the World Health Organization (WHO). After skin disinfection, sterile adhesive pads will be placed on acupoints, and acupuncture needles will be inserted through the adhesive pads. The acupuncture needles used in the study are 40 mm in length and 0.30 mm in diameter and manufactured by Suzhou Hwato Medical Instruments Co. Ltd (Suzhou, China). The following acupoints will be used: DU20 (Baihui), EX-HN3 (Yintang), ST36 (Zusanli), ST37 (Shangjuxu), SP6 (Sanyinjiao), ST25 (Tianshu), and LR3 (Taichong). The depth of insertion is determined based on the standard permissible depth of insertion for each acupoint. DU20 and EX-HN3 will be punctured obliquely 0.5–0.8 cun and 0.2–0.3 cun into the skin, respectively. ST36, ST37, and SP6 will be punctured 1cun into the skin, while ST25 and LR3 will be punctured 1–1.5 cun and 0.5 cun, respectively. Because of hair on the top of head where DU20 is located, no adhesive pad will be placed at this acupoint. Needle manipulation will be applied to achieve De Qi sensation, which is manifested as a numb, distended, and aching sensation. The needles will be maintained for 30 minutes in each session and then removed with clean cotton balls to avoid bleeding. During the treatment period, the needles will be manipulated twice every 10 minutes with intermittent stimulation, and each manual performance will last for 10 seconds. Each patient will be treated with acupuncture 3 times per week and will receive 36 sessions of acupuncture in total during a period of 12 weeks.

### 4.2 Sham acupuncture group

The participants in the sham acupuncture group will receive no skin penetration. After skin disinfection, sterile adhesive pads will be placed on the acupoints. Blunt-tipped placebo needles (25 mm in length and 0.3 mm in diameter) produced by Suzhou Hwato Medical Instruments Co. Ltd (Suzhou, China) will be used in this study. The acupoints are the same as those in the acupuncture group, and similarly, no adhesive pad will be placed on DU20. Hence, we will not needle DU20. The needles will also be maintained for 30 minutes in each session. Each patient will be treated with acupuncture 3 times per week and will receive 36 sessions of acupuncture in total during a period of 12 weeks.

## 5 Outcome assessment

### 5.1 Primary outcome measures

IBS Symptom Severity Score (IBS-SSS) will used to evaluate the degree of severity of symptoms in IBS-D patients. IBS-SSS is an effective and reliable method for assessing the severity of IBS based on the following five parameters: abdominal pain degree, abdominal pain frequency, abdominal distension degree, defecation satisfaction, and influence on life.^[18]^ Each parameter is scored 0 to 100, for a maximum total score of 500. If the score is lower than 75, the patient is considered to be in remission. The mild, moderate, and severe boundary values are 75 to 175, 175 to 300, and above 300, respectively. IBS-SSS will be measured at baseline, in week 4, week 8, and in week 12.

### 5.2 Secondary outcome measures

Visceral Pain Scale will be used to evaluate the severity of visceral pain in IBS-D patients. The scale is designed based on visual analog scale (VAS). A 10-cm horizontal line will be used with a scale from 0 to 100, where 0 indicates the absence of pain and 100 indicates the worst pain imaginable. The participants will rate their level pain by marking on the line. The patients will also receive colonoscopy at baseline and week 13, and the samples of their colonic tissue will be collected to measure miR-199 and TRPV1 levels. Healthy volunteers will only receive one colonoscopy after recruitment.

### 5.3 Data collection and management

A case report form (CRF) has been designed to collect the data of each participant. All clinical observation results will be recorded in the CRF. The information collected will be transcribed to the database which is established based on observation items of the plan. CRFs will be stored in a locked room for at least 10 years in Jiangsu Province Hospital of Traditional Chinese Medicine. The access to data will be restricted to the researchers in this study team.

## 6 Statistical methods

### 6.1 Sample size calculation

There is so far no definite sample size calculation for miR-199 and TRPV1 clinical research. However, based on a clinical trial about TRPV1, there should be almost 20 participants to obtain outcomes with statistical significance.^[8]^ We plan to enroll a total of 40 participants, with 20 in each group.

### 6.2 Statistical analysis

A statistical analysis will be performed using the Statistical Package for Social Sciences (SPSS, version 16.0, SPSS Inc., Chicago, IL) in the Center of Acupuncture Clinical Research, using both the intention-to-treat and per-protocol population analysis. The enumeration data/quantitative data will be expressed as a percentage or proportion, and between-group difference will be analyzed using the chi-square test or Fisher's exact test. The measurement data/qualitative data will be represented as average and standard deviation. When meeting the normal distribution, the data will be analyzed by T test. If not, the Wilcoxon rank sum test will be used to compare the outcome measurements before and after treatment. The *P*-value of .05 or less will be considered significant.

## 7 Discussion

The mechanism of visceral hypersensitivity in IBS is complex. What we know at present is that TRPV1 and miR-199 may play an important role in the development of hypersensitivity in IBS-D patients. Transient receptor potential (TRP) channels have been implicated in the generation (TRPV1, TRPV4, TRPA1)^[19]^ and inhibition (TRPM8) of visceral pain signals in IBS.^[20]^ TRPV1 of IBS patients has been shown to be more sensitive compared to that of healthy volunteers^.^^[21,22]^ Another factor that is likely involved in hypersensitivity is microRNA. TRPV1 is a bona fide target of miR-199. Decreased miR-199 level is correlated with increased TRPV1 expression in the animal model of visceral hypersensitivity.^[10]^

Acupuncture, with its advantages of convenience, satisfactory effects and few adverse reactions, is becoming widely used to treat functional gastrointestinal disorders, especially IBS. Several studies have revealed that acupuncture can attenuate visceral hyperalgesia and alleviate symptoms of IBS-D.^[23,24]^ However, the mechanism which underlies the effect of acupuncture in alleviating hypersensitivity remains unclear. Hence, we designed this randomized, placebo-controlled clinical trial to test our hypothesis.

In this study, sham acupuncture is used as a control to eliminate potential placebo effect of acupuncture. In acupuncture research, various types of sham acupuncture have been used, including minimal acupuncture on non-TCM points, sham laser on acupoints, and placebo needles.^[25]^ In our trial, in order to help maximize blinding of participants, a pragmatic placebo needle (similar to the Streitberger design^[26]^) will be used. The placebo needle in the sham group is similar in appearance to the conventional needle in the acupuncture group, but has a blunted tip and will not penetrate the skin.

There are still limitations that should be noted. First, due to the discomfort and risk associated with receiving colonoscopy twice, recruitment will be considerably difficult. Second, due to the long duration of our study design extending beyond the general treatment of 6 weeks, the dropout rate may be higher. In order to minimize potential dropout, we have established guidelines and training for research staff to establish good communication-relationships with the participants. Moreover, characteristics of the dropouts will be extracted and analyzed. Third, due to difficulty in placing an adhesive pad on the head, we have decided not to needle DU20 (Baihui) in the sham acupuncture group. Hence, this may generate a performance bias.

In conclusion, the results of this trial are expected to not only provide clinical evidence of the effectiveness of acupuncture in treating IBS-D, but also demonstrate that the effect is achieved by increasing the expression of microRNA-199 in the colon and reducing the activation of TRPV1.

## Acknowledgments

We acknowledge Digestive Endoscopy Center in Jiangsu Province Hospital of TCM for recruiting patients and colonoscopy. We also appreciate the help and efforts of all research staff participating in this trial.

## Author contributions

Authorship: Li-Xia Pei, Hao Chen and Jian-Hua Sun conceived this trial and participated in the design of the trial. Jing Guo, Lu Chen and Xiao-Liang Wu were responsible for planning to draft. EunMee Yang and Trine Hammer revised the manuscript. Wan-Li Xu and Sheng-Jie Weng are monitors of this study. All authors read this manuscript and approved the publication of this protocol.

**Conceptualization:** Lixa Pei, Hao Chen, Jianhua Sun.

**Project administration:** Jianhua Sun.

**Supervision:** Wanli Xu, Shengjie Weng.

**Validation:** Wanli Xu, Shengjie Weng.

**Visualization:** Wanli Xu, Shengjie Weng.

**Writing – original draft:** Jing Guo, Lu Chen, Xiaoliang Wu.

**Writing – review & editing:** EunMee Yang, Trine Hammer.

## Supplementary Material

Supplemental Digital Content
