# Supplementary material for: Effect of acupuncture and its influence on visceral hypersensitivity in IBS-D patients: Study protocol for a randomized controlled trial
Source: Medicine (Baltimore). 2018 May 25;97(21):e10877. doi: 10.1097/MD.0000000000010877 (PMC6392752; doi:10.1097/MD.0000000000010877)
Supplement: Supplemental Digital Content [file medi-97-e10877-s001.doc]

**Ethical approval**

**Document number：**2016NL-078-03

**Project name：**Exploring the intervention mechanism of acupuncture on visceral hypersensitivity of IBS-D patients based on microRNA199-TRPV1：study protocol of a randomized controlled trial

**Project source：**Provincial Natural Science Foundation of Jiangsu

**Research unit：**Jiangsu Province Hospital of TCM

**Project leader：**Li-xia Pei

**Category of review：**Application for review **Method of review：**Quick review

**Date of review:** Mar. 04, 2017

**Review committee：**Luo Fu

**Review documents:** Modified clinical research program, Version number: 2.0, Version date: Feb. 16, 2017;Revised informed consent, Version number: 3.0., Version date: Mar. 04, 2017;Revised recruitment matierials,Version number:3.0,Version date:Mar.04,2017.

**Review opinion：**

- According to the Ministry of Health "biomedical research involving human ethics review (Trial)" (2007), SFDA "drug clinical trials quality management standards" (2003), "the clinical trial of medical devices regulations" (2004), WMA "ethical principles of the Helsinki declaration" and "human CIOMS international ethical guidelines for biomedical research", by the ethics review committee, agreed with the clinical research plan, in accordance with the approved informed consent of the study book, recruitment materials.
- Please follow the GCP principles and follow the program approved by the ethics committee to carry out clinical research and protect the health and rights of the subjects.
- Before start of the study, applicants should complete the clinical trial registration
- In the process of research, if any modifications are made to the main researchers, clinical research plan, informed consent, recruitment materials, etc., applicants are required to submit amendments to the review application.
- If a serious adverse event occurs, the applicant is requested to submit a report on the adverse event in time
- According to the annual / periodical review frequency stipulated by the ethics committee, the applicant will submit the research progress report 1 months before the deadline. The sponsor shall submit a summary report of the research progress of each center to the leader's unit ethics committee. Please submit a written report to the ethics committee in time when there is anything that may significantly affect the test, or increase the risk of the subject.
- If it occurs any event, such as not meeting the inclusion criteria, meeting the exclusion criteria of subjects, without participants withdrew on the exit provisions from the study, giving the wrong treatment or dose, given scheme prohibited coadministration, Infringement of rights and interests and violation of GCP principle, sponsor/supervisor /researcher are supposed to bid the ethics committee a report submitted against the plan.
- If he applicant suspends or terminates the clinical study in advance，please submit the sustentation / termination research report in time.
- When completing the clinical study, the applicant is asked to submit the research report, and summarize the summary report of the findings and conclusions.

**Contacts：**Jing Wu 025-86560515

**Signature of president:** Ming-hua Wu

**Ethics Committee：**Ethics Committee of the Affiliated Hospital of Nanjing University of Chinese Medicine (Jiangsu Province Hospital of TCM)

**Date of approval:** Mar. 04, 2017

Department of Finance of Jiangsu Province

File

 Department of Science and Technology of Jiangsu Province

Su Cai Jiao [2016] 88

Department of Finance of Jiangsu Province

Department of Science and Technology of Jiangsu Province

Notice of 2016 Provincial Natural Science Foundation of Jiangsu (Special Fund For Youth Science and Technology Talents)

To the relevant Municipal, County Finance Bureau ,Science and Technology Bureau, and the relevant provincial units:

Notice of 2016 Provincial Natural Science Foundation of Jiangsu (special fund for youth science and technology talents) is given to you.In the attachment are funds and projects. Correspondingly "provincial natural science foundation " budget expenditure index will be increased in 2016 (revenue and expenditure classification subject code 2060203).

Please strictly follow the relevant regulations on the management of scientific and technological funds, such as the “Provisional Measures for the Management of Funds for Jiangsu Provincial Science and Technology Special Funds”. Strengthen the organization coordination and management of projects according to the requirements and project contracts to ensure the science and technology funds earmarked to the project matching funds, raised funds in a timely manner in full, completion of the project on time and achieving early results and benefits. Each unit in the project should report to Jiangsu Province Department of Finance and Jiangsu Province Department of Science and Technology about the use of funds in the end of the year. And then the two department will organize experts to examine and assess the implementation of the project and the use of funds.

Department of Finance of Jiangsu Province

Department of Science and Technology of Jiangsu Province

2016/06/28

**Project Number:** BK20161083

**Project Name:** Exploring the intervention mechanism of acupuncture on visceral hypersensitivity of IBS-D patients based on microRNA199-TRPV1

**Undertaking Unit：**Jiangsu Province Hospital of TCM

**Competent Department：**Provincial Administration of Traditional Chinese Medicine

**Project Leader：**Li-xia Pei

**Finish Time：**2019

**Funds :**200，000
